# A SARM a Day Keeps the Weakness Away: A Computational Approach for Selective Androgen Receptor Modulators (SARMs) and Their Interactions with Androgen Receptor and 5‑Alpha Reductase Proteins

**DOI:** 10.1021/acsomega.5c02504

**Published:** 2025-07-18

**Authors:** Mustafa Munir Mustafa Dahleh, Silvana Peterini Boeira, Hecson Jesser Segat, Gustavo Petri Guerra, Marina Prigol

**Affiliations:** † Laboratory of Pharmacological and Toxicological Evaluations Applied to Bioactive Molecules − LaftamBio − Federal University of Pampa, Itaqui, CEP 97650-000, RS, Brazil

## Abstract

This study examines the molecular interactions of selective androgen receptor modulators (SARMs) with the androgen receptor (AR) and 5-alpha reductase II (5αRII), highlighting their potential as dual-action pharmacological candidates, using molecular modeling techniques to evaluate their primary interactions, providing valuable insights into conformational stability and ligand-induced changes and enabling rational analysis of SARMs with optimized pharmacological profiles. Employing molecular docking, density functional theory (DFT), and molecular dynamics simulations, we analyzed the binding affinities and conformational stability. Between all eight SARMs tested, 4′-[(2*S*,3*S*)-2-ethyl-3-hydroxy-5-oxopyrrolidin-1-yl]-2′-(trifluoromethyl)­benzonitrile (Sarm2f) demonstrated exceptional stability and binding affinity with critical interactions at key AR residues such as Asn705, Glu711, Arg752, and Thr877. The inclusion of fluorinated groups enhances hydrogen bonding through dipole induction, improving the binding dynamics. Additionally, Sarm2f interacts with small hydrophobic pockets around the 5-oxopyrrolidine ring, further stabilizing its conformation. Also, (17α,20*E*)-17,20-[(1-methoxyethylidene)­bis­(oxy)]-3-oxo-19-norpregna-4,20-diene-21-carboxylic acid methyl ester (YK11) exhibited compelling interactions with both AR and 5αRII, characterized by a tetracyclic steroidal nucleus that enhances its androgenic activity. Structural modifications, including a double bond at the C20 position in YK11, improve stability and prolong interactions with the AR. While Sarm2f shows lower root-mean-square deviation (RMSD) values, indicating rigidity, the slight flexibility of YK11 may allow for a broader range of interactions. These findings emphasize the importance of advanced computational methods in optimizing SARMs by demonstrating how specific chemical modifications affect binding affinity and selectivity for AR and 5αRII, thereby aiding the development of safer and more effective pharmacological agents for androgen-related conditions.

## Graphical abstract

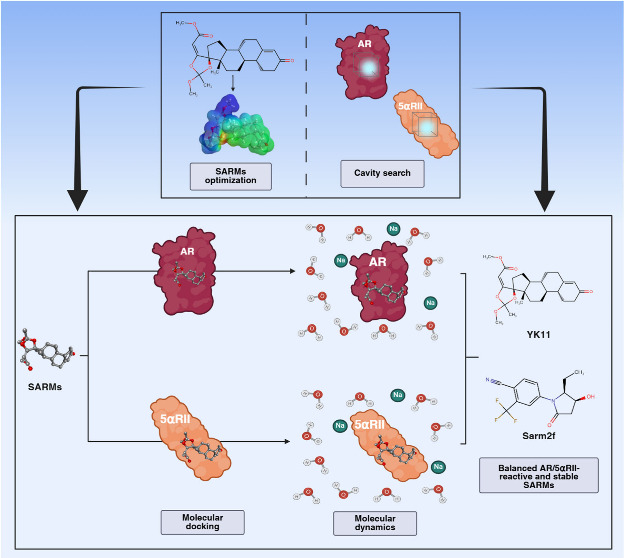

## 1 Introduction

Selective Androgen Receptor Modulators (SARMs) have emerged as a promising class of pharmacological agents, attracting considerable interest due to their anabolic properties.^1^ First developed in the late 1990s, SARMs were designed to selectively activate the Androgen Receptor (AR) similar to dihydrotestosterone (DHT), aiming to enhance anabolic properties in specific tissues.^2,3^ Unlike traditional anabolic androgenic steroids (AAS), which often produce widespread adverse effects due to their broad action across multiple tissues, SARMs were engineered for a more targeted approach. This selectivity allows them to focus primarily on skeletal muscle and other desired tissues, thereby reducing undesirable impacts on the prostate and other nontarget tissues^3,4^

Primarily nonsteroidal compounds, such as ostarine (GTX-024) and ligandrol (LGD-4033)^5^ with a few steroidal exceptions like YK11,^6^ SARMs are designed to bind with high affinity and specificity to the ligand-binding domain (LBD) of the AR.^7^ The resulting conformation of the AR complex promotes the dissociation of heat shock proteins (Hsp), particularly Hsp90, in the cytosol, allowing the translocation of the AR-ligand complex to the cell nucleus.^8,9^ In the nucleus, dimerized ARs bind to androgen response elements (AREs) in the promoter regions of target genes, such as the creatine kinase (CKM) gene,^10,11^ insulin receptor (INSR) gene,^12,13^ and also the mammalian target of rapamycin (mTOR),^14,15^ initiating the transcription of genes responsible for tissue growth. This DNA binding is facilitated by some interactions, such as Arg752 forming a hydrogen bond with the O3 atom (ketone) of the steroid ligand, within the transcription factor, stabilizing the protein–DNA interaction.^16–18^

Additionally, AR binding can induce various receptor conformations, leading to distinct coregulator profiles that modulate gene expression in a tissue-specific manner.^19^ Co-activators such as nuclear receptor coactivator 1 (NCoA-1) and nuclear receptor coactivator 2 (NCoA-2),^20,21^ corepressors like nuclear receptor corepressor 1 and 2 (NCoR1 and NCoR2),^22^ and silencing mediator for retinoid and thyroid-hormone receptors (SMRT),^23^ interact with AR in unique ways depending on the specific SARM or testosterone derivative, resulting in a finely tuned transcriptional response. These interactions typically occur after metabolic processes involving 5-alpha-reductase, which converts, for example, testosterone into DHT by reducing the double bonds between the fourth and fifth carbon atoms.^24^ In this sense, it is important to consider whether SARMs undergo similar metabolic transformations by 5-alpha-reductase, given that their effects on AR are similar to those of DHT.

5α-reductase II (5αRII) catalyzes the conversion of testosterone into DHT, a more potent androgen, playing a crucial role in regulating androgenic activity.^24–26^ Within its active site, residues like Glu57 facilitate testosterone binding and NADPH reduction.^27,28^ Competitive inhibitors such as finasteride target this site, suppressing 5αRII activity.^28^ Unlike testosterone, SARMs are not converted by 5αRII, maintaining anabolic potency without DHT-associated side effects like prostatic hypertrophy.^5,29,30^ Some SARMs may even exhibit finasteride-like inhibition of 5αRII, potentially increasing testosterone bioavailability and influencing the HPG axis.^31^

The growing interest in SARMs for physical performance is due to their anabolic benefits similar to AAS, but with a better safety profile.^3^ However, understanding their long-term efficacy requires further research. Molecular modeling is crucial for examining interactions of SARMs with AR and 5αRII enzymes. This study aims to investigate these key interactions through molecular modeling to assess their pharmacological effects, offering insights into conformational stability and ligand-induced changes and optimizing SARMs’ pharmacological profiles.

## 2 Materials and Methods

### 2.1 Acquisition, Selection and Geometric Optimization of SARMs Structures

Control molecules were obtained from cocrystallized structures sourced from the Cambridge Crystallographic Data Centre (CCDC) or the Protein Data Bank (PDB): DHT (PDB ID: 4OEA), testosterone (PDB ID: 2AM9), and finasteride (CCDC ID: 1294659). For the experimental design, SARMs were selected according to three overarching criteria: documented nonclinical human use, current commercial availability or advanced commercialization stage, and a paucity of published mechanistic data on their interactions with AR and 5αRII. Based on that, we selected these compounds, ostarine, ligandrol (LGD-4033), testolone (RAD-140), andarine (S-4), S-23, S-101479, Sarm2f, and YK11, for their widespread nonmedical use as muscle-building and fat-loss agents with reportedly fewer side effects than traditional anabolic steroids (Figure 1).^32^ These eight molecules are among the most frequently self-administered for performance enhancement and aesthetic purposes, yet they remain poorly characterized with respect to their binding profiles to AR and 5αRII.^32^ By focusing on compounds with emerging, albeit limited, evidence of AR affinity and potential 5αRII inhibition, our study aims to address a critical gap in the current literature. All ligands were prepared following the docking protocol outlined below, ensuring a consistent and standardized framework for comparative in silico analysis.^3^

**Figure 1:**
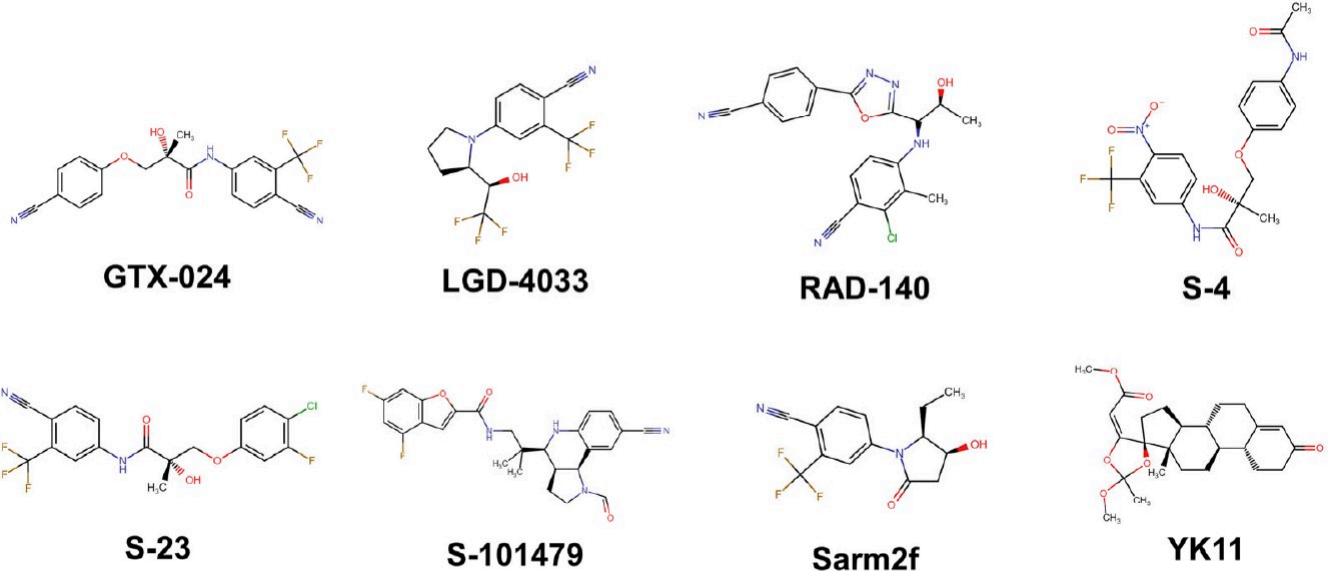
SARMs structures selected for affinity testing with AR, and 5αRII.

The selected SARMs included ostarine (GTX-024, IUPAC: (2*S*)-3-(4-cyanophenoxy)-*N*-(4-cyano-3-trifluorophenyl)-2-hydroxy-2-methylpropanamide), ligandrol (LGD-4033, IUPAC: 4-((*R*)-2-((*R*)-2,2,2-trifluoro-1-hydroxyethyl)­pyrrolidin-1-yl))-2-trifluoromethylbenzonitrile), testolone (RAD-140, IUPAC: 2-chloro-4-((1*R*,2*S*)-1-(5-(4-cyanophenyl)-1,3,4-oxadiazol-2-yl)-2-hydroxypropylamino)-3-methylbenzonitrile), andarine (S-4, IUPAC: 3-(4-acetylamino-phenoxy)-2-hydroxy-2-methyl-*N*-(4-nitro-3-trifluoromethyl-phenyl)-propionamide), S-23 (IUPAC: (*S*)-*N*-(4-cyano-3-trifluoromethyl-phenyl)-3-(3-fluoro-4-chlorophenoxy)-2-hydroxy-2-methyl-propanamide), and S-101479 (IUPAC: *N*-(2-((3a*S*,4*S*,9b*S*)-8-cyano-1-formyl-2,3,3a,4,5,9*b*-hexahydro-1*H*-pyrrolo^3,2^quinolin-4-yl)-2-methylpropyl)-4,6-difluorobenzofuran-2-carboxamide), Sarm2f (IUPAC: 4′-[(2*S*,3*S*)-2-ethyl-3-hydroxy-5-oxopyrrolidin-1-yl]-2′-(trifluoromethyl)­benzonitrile), and YK11 (IUPAC: (17α,20*E*)-17,20-[(1-methoxyethylidene)­bis­(oxy)]-3-oxo-19-norpregna-4,20-diene-21-carboxylic acid methyl ester) (Figure 1).

The initial molecular geometries of SARMs were obtained through protomeric and conformational sampling using CREST software v2.12. All subsequent density functional theory (DFT) calculations were performed by using the quantum chemistry package ORCA v5.0.3. The most stable conformer underwent complete geometric optimization based on DFT at the B3LYP OPT/def2-TZVP level, incorporating the conductor-like polarizable continuum model (CPCM) to simulate aqueous environments. The resulting stable geometries of conformers, having no imaginary vibrational modes, were considered to be true energy minima. These results formed the basis for vertical-transition-time-dependent density functional theory (TD-DFT) calculations at the same level, which were performed with 100 states to select the most representative ones.

### 2.2 Density Functional Theory (DFT) Calculations

DFT calculations were conducted using the quantum chemistry software ORCA v5.0.3.^33^ Initial molecular geometries for SARMs were derived through conformational sampling via the semiempirical method CREST.^34^ Subsequently, the most stable conformers underwent further optimization employing the M06-L/def2-SVP level of theory within a Laplacian operator model [*E*_XC_ = ∫*F*(ρ,∇ρ,∇^2^ρ) d*r*] (Kulkarni and Truhlar, 2011),^100^ and the Conductor-like Polarizable Continuum Model (CPCM)^35^ for implicit solvation. The molecular electrostatic potential *V*(*r*) produced across the molecule due to the combined effect of negative and positive charges corresponding to electrons and nuclei is given by the expression [ $V_{\left(r\right)}=\sum A=\frac{Z_{A}}{\left|R_{A}^{\to }-r^{\to }\right|}-\int \frac{\rho \left(r^{\to }\right)}{\left|r^{\to }-r^{\to }\right|}$ ], where *Z*_*A*_ is the charge on nucleus *A*, and ρ­(*r* ^→^) is the electrostatic density of the molecule. Finally, the molecular electrostatic potential was calculated and plotted using MoCalc2012 v4.2.0.1 with Jmol extension.^36^ This process generated Mulliken Atomic Charges, a critical tool for interpreting a molecule’s chemical reactivity, particularly toward electrophilic and nucleophilic attacks, enabling the assessment of direct or indirect interactions with other molecules.

### 2.3 Cavity Search Studies

The CavityPlus Web server software was employed to identify cavities and their binding sites, predict allosteric sites, assess the potential for covalent ligand-binding events, and generate pharmacophore measurements.^37^ Initially, 3D protein models were obtained by inputting the corresponding Protein Data Bank (PDB) code or SIB Swiss Model Archive (SSMA) ID: AR (PDB-1T7T),^38^ (PDB-2PIW),^39^ (PDB-2AMA),^5,16^ αRII (PDB-7BW1),^28^ (SSMA-ma-ib3wq).^40^ The cavity search utilized specific parameters: a minimum depth of 8 Å, a maximum abstract limit of 1,500 Å^3^, a separate maximum volume of 6,000 Å^3^, a minimum abstract depth of 2 Å, a minimum value limit of 100 Å^3^, and a minimum score threshold of 1.5. The CavityPlus CAVITY module was used to identify and rank potential binding sites on AR and 5αRII based on four main metrics: predicted maximal p*K*_d_, predicted average p*K*_d_, DrugScore, and druggability. predicted maximal p*K*_d_ reflects the highest theoretical binding affinity within a cavity, while the predicted average p*K*_d_ estimates the mean affinity across various probes, indicating overall ligandability. DrugScore integrates physicochemical and geometric features (e.g., hydrophobicity, enclosure, and hydrogen bonding potential) to quantify the pocket suitability for small-molecule binding. Based on these values, druggability is assigned as high, medium, or low. Cavities were prioritized according to their predicted maximal p*K*_d_ and DrugScore and further filtered by druggability category, average p*K*_d_, and volume, ensuring that selected sites were not only theoretically favorable but also consistently ligandable and sufficiently spacious. Crystallized protein structures (AR and 5αRII) were selected based on a combination of binding reactivity (evaluated via native ligands) and structural resolution.

### 2.4 Molecular Docking Simulations

Molecular docking analyses were conducted to investigate the potential interactions between SARMs and their major physiological targets within the most druggable cavities identified through cavity search studies. Crystallized proteins were obtained from the PDB: AR (PDB-1T7T), and 5αRII (PDB-7BW1), as early described. The 2D structures of SARMs were cocrystallized using quantum chemistry models based on DFT, and other conformations were directly obtained from their crystallized geometries in the Cambridge Crystallographic Data Center (CCDC), as also delineate previously. To validate the accuracy of the docking protocol, redocking was performed using the native ligand of each protein, and the resulting positions were compared to the crystal structure positions by calculating the root-mean-square deviation (RMSD) values. Validation demonstrated an RMSD within the acceptable range of ≤ 2 Å,^41^ with AR–DHT = 0.37 Å and 5αRII–finasteride = 0.56 Å. During the docking simulations, the ligands were treated as flexible while the receptors were kept rigid (Gasteiger charges) using AutoDockTools.^42^ The analysis of the formed complexes involved the removal of the native ligand from the protein pocket sites. Docking simulations were conducted using AutoDock Vina software,^43^ with grid box dimensions derived from the coordinates obtained from the CavityPlus Web server for the most reactive site of each protein, with an exhaustiveness setting of 100. For the binding pocket analyses, DHT was used as the reference ligand for AR interactions, while finasteride and testosterone served as reference ligands for 5αRII binding assessments. Subsequent analysis of the docking results was conducted using AutoDock Vina and Discovery Studio Visualizer. For each docked pose, 10 independent replicates were performed using different random seeds to account for the potential variability in the docking protocol. The docking output included estimated binding free energies (Δ*G*_bind_, in kcal/mol) and their corresponding residue-level interactions. During 3D visualization, all types of molecular interactions were considered, with the exception of van der Waals contacts, which are explicitly reported only when they are particularly relevant.

### 2.5 Molecular Dynamics Simulations

The GROMACS engine^44^ was employed for conducting all molecular dynamics simulations, specifically focusing on ligand–protein complexes within explicit water. Initial poses for these simulations were derived from prior molecular docking investigations targeting the most druggable cavities within AR and 5αRII protein, with SARMs, DHT, testosterone, and finasteride.

For protein preparation, the AMBER99 force field^45^ was employed, and ligand topologies were generated using the PRODRG tool.^46^ The TIP3P water model^47^ was applied, and a triclinic water box was used for the protein–ligand complex. Prior to starting the molecular dynamics simulations, system minimization was performed using the steepest descent algorithm with 5000 steps, followed by NVT/NPT equilibrations. The simulations were conducted in the presence of 0.15 M NaCl ions under constant temperature (298 K) and pressure (1.0 bar) conditions. Each simulation generated approximately 5000 frames, with a duration of 50 ns, in both AR^48,49,5^ and αRII^50,51^ proteins. Through each simulation, Root-Mean-Square Deviation (RMSD) and Root-Mean-Square Fluctuation (RMSF) data were obtained to evaluate the stability and changes over time in the complex formed with each protein. DHT molecule served as a fluctuation control model in AR fluctuations, with testosterone and finasteride in 5αRII fluctuations. Approximately 5000 frames were generated for each simulation, with each simulation running for 50 ns.

## 3 Results and Discussion

### 3.1 Analysis of the Electrostatic Potential of SARMs

Common structural features and electrostatic potential distributions significantly influence their interactions, impacting their binding affinity and inhibitory capabilities.^52^ Analysis of the electrostatic potential data (eV), via Mulliken atomic charge (Table 1), provides significant insights into biochemical mechanisms and efficacy regarding several SARMs and physiological targets, such as AR and 5αRII enzymes. All the molecules under analysis, including GTX-024, LGD-4033, RAD-140, S-4, S-23, S-101479, Sarm2f, and YK11, share some fundamental characteristics that drive their interactions with AR and 5αRII enzymes. These molecules typically contain aromatic rings, which facilitate hydrophobic interactions within the AR^53^ and/or 5αRII^26^ binding pocket, stabilizing the ligand–receptor complex. They often feature nitrogen and oxygen atoms capable of engaging in hydrogen bonding with AR amino acid residues, further stabilizing these substances,^39^ as expected given their designations as SARMs.

**Table 1:**
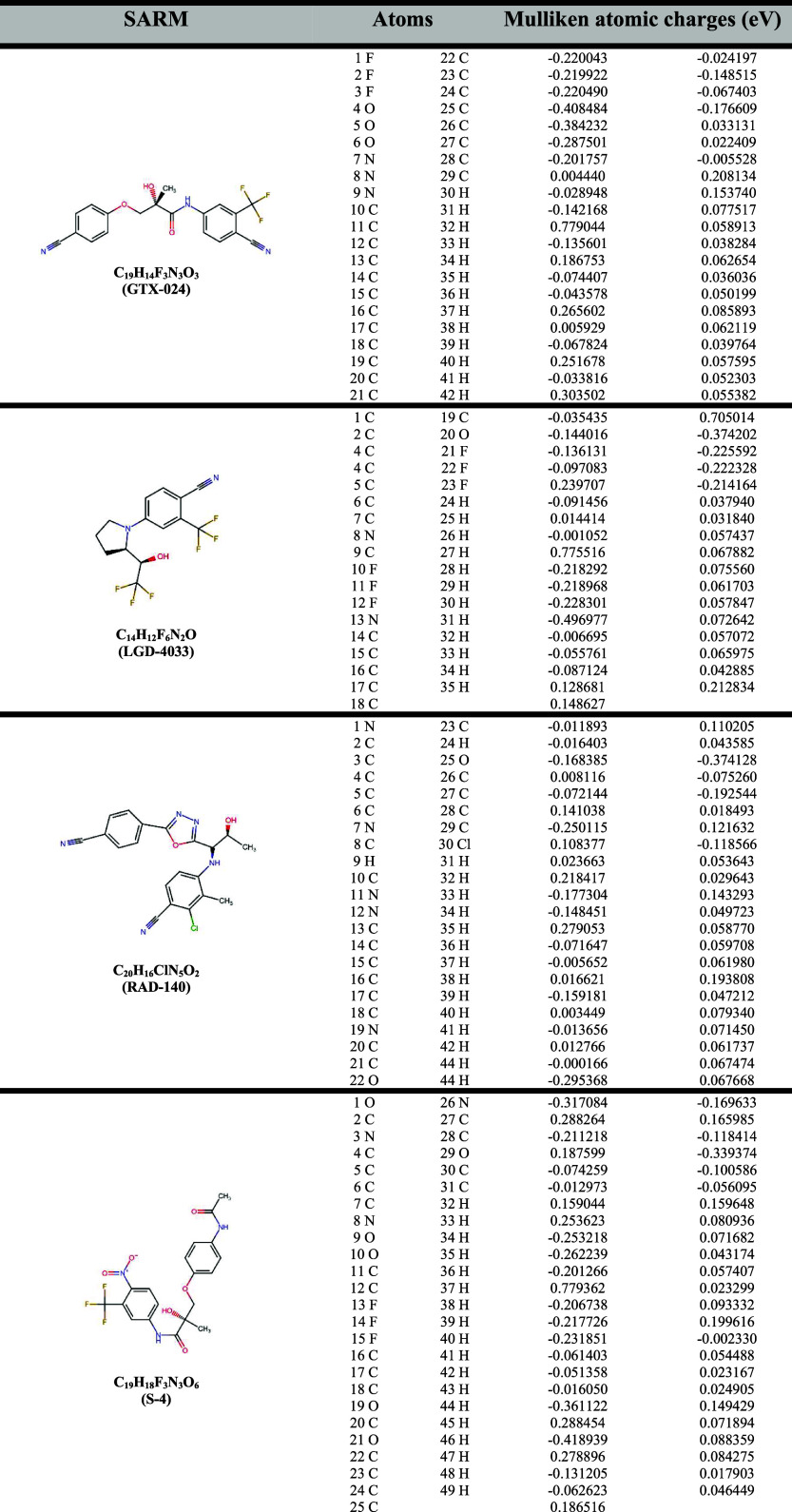

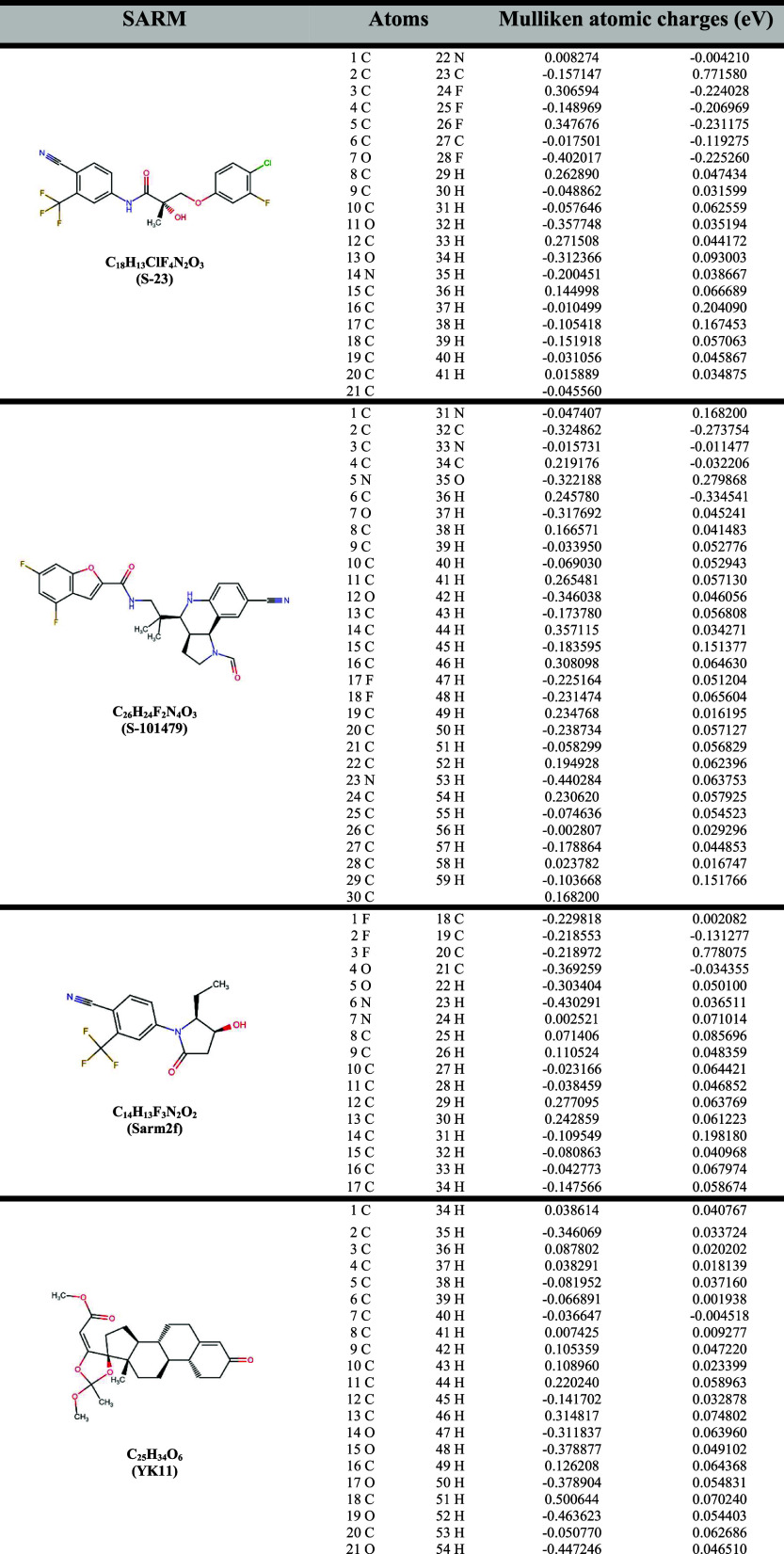

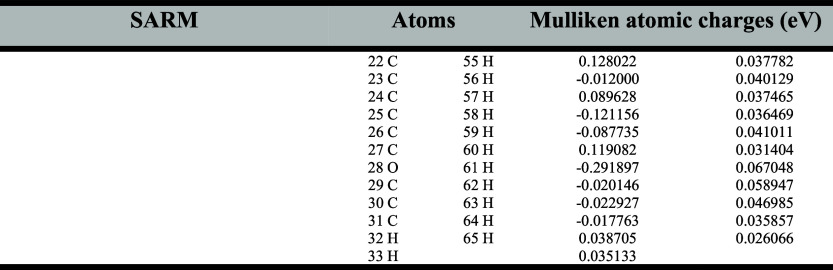
Mulliken Atomic Charges from DFT-Based Calculation Analysis of SARMs

In the evaluation of the binding interactions of various SARMs with AR and 5αRII, key structural features play a significant role. SARMs such as GTX-024 (Figure 2A), LGD-4033 (Figure 2B), RAD-140 (Figure 2C), and S-4 (Figure 2D) are characterized by the presence of fluorine atoms, which enhance their electronegativity and lipophilicity.^54,55^ The fluorine atoms in these compounds exhibit electrostatic potentials around −0.2 eV, indicating a strong nucleophilicity. For instance, in LGD-4033, fluorine atoms show potentials of −0.218292 eV (10F), −0.218968 eV (11F), and −0.228301 eV (12F). These fluorine atoms, combined with nucleophilic nitrogen and oxygen atoms, contribute significantly to their binding affinity and selectivity for AR.^56^

**Figure 2:**
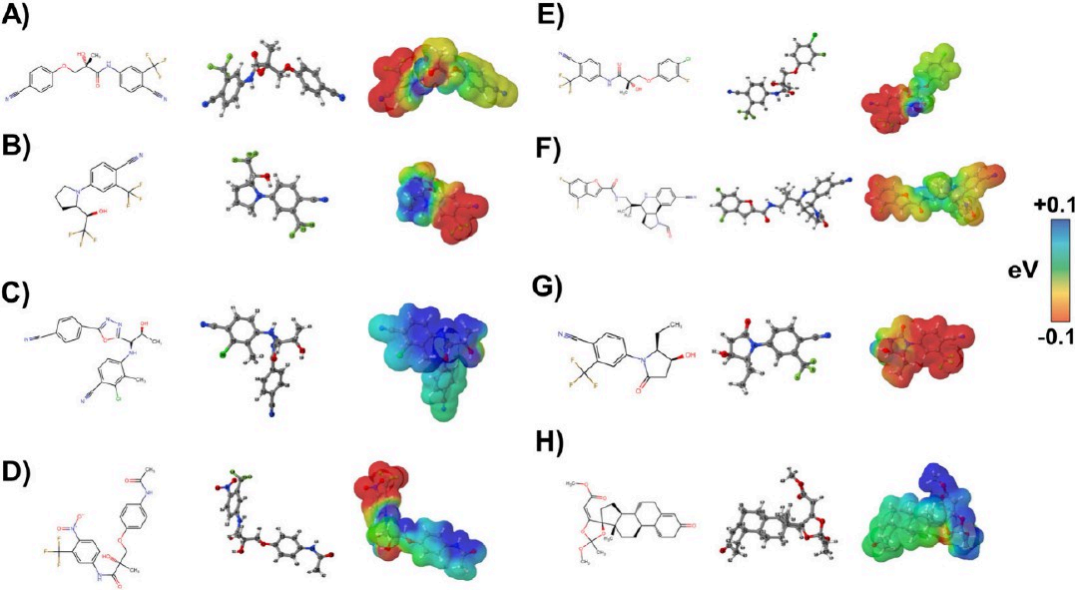
Computed molecular electrostatic potential (MEP) maps of SARMs using an M06L/def2-SVP theoretical model, represented in electron volts (eV). Areas with more negative values (red) indicate higher susceptibility to nucleophilic attack, while areas with more positive values (blue) indicate higher susceptibility to electrophilic attack. The SARMs analyzed are represented in (A) GTX-024; (B) LGD-4033; (C) RAD-140; (D) S-4; (E) S-23; (F) S-101479; (G) Sarm2f; (H) YK11.

S-23 (Figure 2E) and S-101479 (Figure 2F) also display notable electrostatic characteristics, with nucleophilic oxygen and fluorine atoms enhancing hydrogen bonding and hydrophobic interactions within the AR binding site.^57^ Their complex, multiring structures optimize their fit into the AR binding site, increasing the strength of interaction.^58^ For example, S-23 features oxygen atoms with potentials like −0.402017 eV (O7) and −0.357748 eV (O11), indicating strong nucleophilic regions that may inhibit 5αRII by competing with testosterone and sterically hindering its access to the enzyme active site.^25^

Sarm2f (Figure 2G) is another compound with relevant properties, featuring fluorine atoms with potentials of −0.229818 eV (1F) and −0.218553 eV (2F), alongside oxygen atoms with potentials of −0.369259 eV (4O). These attributes enhance its interaction with the AR binding pocket and suggest potential inhibition of 5αRII activity, thereby reducing DHT production.^25,59,60^ YK11 (Figure 2H), notable for its dual function as a myostatin inhibitor,^6,59^ also shares structural features such as aromatic rings and nucleophilic atoms. Electrostatic potential data for YK11 reveal significant nucleophilic regions, including oxygen atoms with potentials of −0.311837 eV (O14) and −0.378877 eV (O15). These features enhance both AR binding and potential interactions with 5αRII. YK11 dual activity not only promotes AR binding but also inhibits myostatin, which contributes to increased muscle growth, and its molecular shape and size may impact the inhibition of 5αRII.

Overall, SARMs such as LGD-4033 and RAD-140 exhibit a high binding affinity primarily for AR due to their strong nucleophilic regions. In contrast, S-4, S-23 and S-101479 are more likely to interact with 5αRII, potentially leading to competitive inhibition. Of note, Sarm2f and YK11 show dual potential to engage with both AR and 5αRII, underscoring their versatility in therapeutic applications. These compounds integrate various structural features, including aromatic rings, nucleophilic atoms, and fluorine atoms, which influence their interactions with AR and 5αRII. We will further investigate these interactions by studying these proteins in conjunction with the SARM molecules to confirm the potential site reactions identified.

### 3.2 AR and 5αRII Cavity Reactivity and Binding Affinity with SARMs

#### 3.2.1 Androgen Receptor (AR)

To evaluate the reactivity of AR, we selected three crystallized structures of the AR obtained from the PDB with codes 1T7T, 2AMA, and 2PIW. For each protein model, we identified the highest reactivity cavity sites (CAVs) (Table S1, available in the Supporting Information).

In the 1T7T structure (Figure 3A), we identified 8 CAVs. The analysis revealed that CAV1 exhibited the highest druggability with a maximum p*K*_d_ of 10.37, an average p*K*_d_ of 6.93, and a DrugScore of 1392, categorizing it as a highly druggable site (++). Similarly, CAV2 and CAV3 showed substantial druggability with maximum p*K*_d_ values of 10.90 and 9.45, average p*K*_d_ values of 6.36 and 5.86, and DrugScores of 301 and 26, respectively, both categorized as medium druggable sites (+). The remaining cavities (CAV4 to CAV8) demonstrated significantly lower druggability with negative DrugScores and lower p*K*_d_ values, indicating weak druggability (−). In the 2AMA structure (Figure 3B), we identified 5 CAVs. CAV1 stood out with a maximum p*K*_d_ of 10.94, an average p*K*_d_ of 6.97, and a DrugScore of 1655, marking it as a highly druggable site (++). CAV2 and CAV3 also presented considerable druggability with maximum p*K*_d_ values of 10.03 and 9.39, average p*K*_d_ values of 6.06 and 5.84, and DrugScores of 39 and 81, respectively, both falling into the medium druggable category (+). CAV4 and CAV5 showed lower druggability with negative DrugScores, indicating weak druggability (−). For the 2PIW structure (Figure 3C), 8 CAVs were identified. CAV1 displayed the highest druggability with a maximum p*K*_d_ of 10.29, an average p*K*_d_ of 6.92, and a DrugScore of 1591, categorizing it as highly druggable (++). Interestingly, CAV3, despite a maximum p*K*_d_ of 8.81 and an average p*K*_d_ of 5.64, had a negative DrugScore of −71, suggesting medium druggability (+) but indicating potential challenges in binding efficacy. The remaining CAVs in this structure exhibited weak druggability with negative DrugScores and lower p*K*_d_ values, particularly for CAV2, CAV4, CAV5, CAV6, CAV7, and CAV8, which had significantly lower druggability ratings.

**Figure 3:**
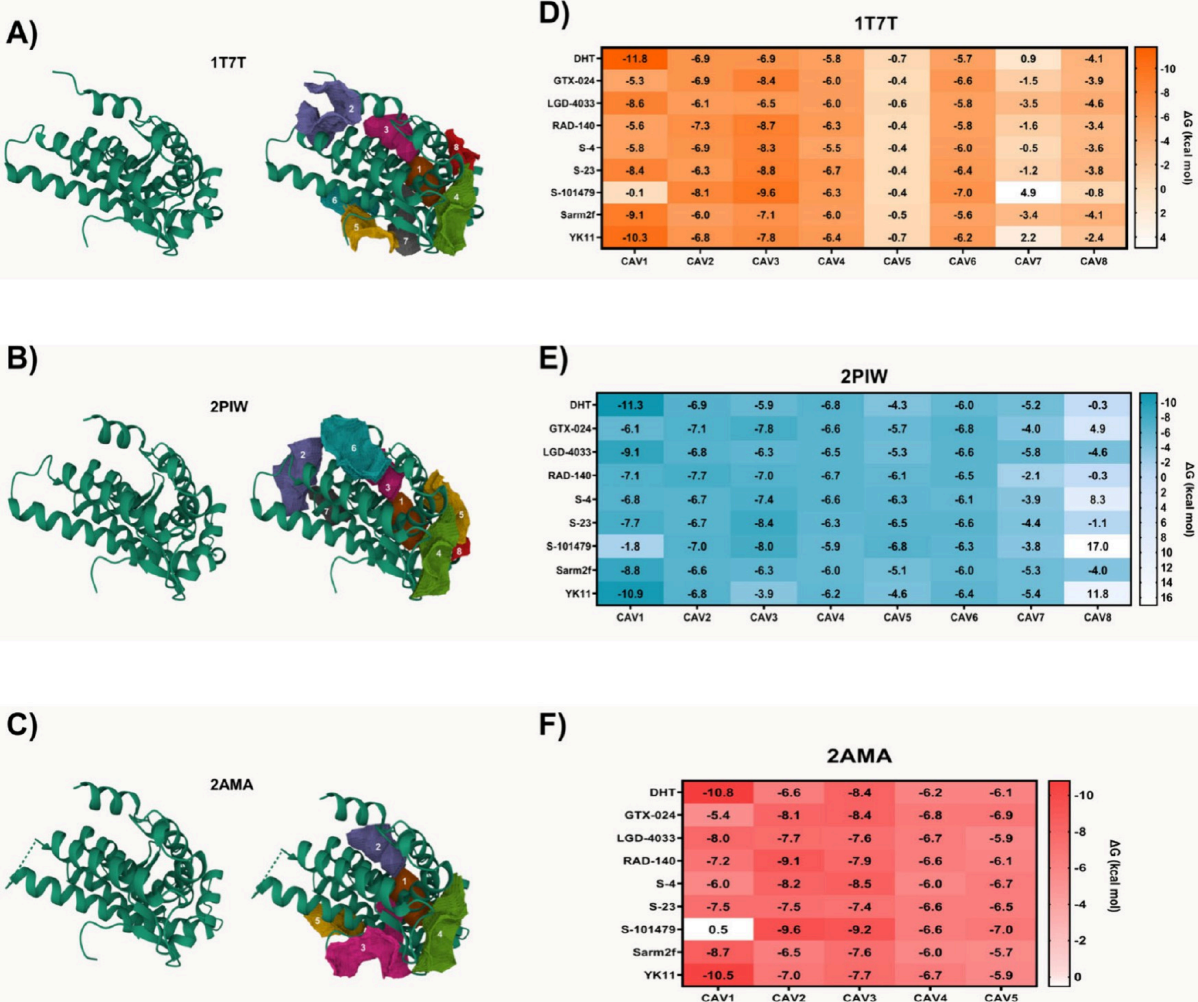
Analysis of the most reactive cavities (CAV) in different AR species (A–C), with data obtained using the CavityPlus Web server. Molecular docking was used to analyze potential affinity between the identified cavities in each AR species and SARMs (D–F), with data obtained through AutoDockVina and Discovery Studio Visualizer.

Molecular docking tests showed that the highest affinity between DHT and SARMs predominantly occurred in the CAVs with the highest druggability in all three of the tested conformations. Specifically, CAV1 in all models displayed the greatest reactivity with the native molecule, DHT. The Δ*G*_bind_ of DHT with CAV1 was −11.8 kcal/mol for 1T7T, −11.3 kcal/mol for 2PIW, and −10.8 kcal/mol for 2AMA. The reactivity patterns observed in the heatmaps (Figure 3D–F) further corroborate these findings. In 1T7T (Figure 3D), 2PIW (Figure 3E), and 2AMA (Figure 3F), the binding affinities for DHT and various SARMs across the identified CAVs consistently highlight CAV1 as the most reactive and druggable site, showing the lowest binding free energy values. Notably, LGD-4033, Sarm2f, and YK11 demonstrated a high affinity for CAV1 across all three tested conformations. The Δ*G*_bind_ values ranged from −8.0 to −9.1 kcal/mol for LGD-4033, −8.7 to −9.1 kcal/mol for Sarm2f, and −10.3 to −10.9 kcal/mol for YK11. While S-101479 does not appear to interact significantly with the primary AR site, its potential anabolic effects should not be discarded, considering its possible inhibitory impact on 5αRII proteins.

This consistency across different AR conformations suggests that targeting CAV1 could be a robust strategy for developing SARMs. Therefore, CAV1 will be designated as the primary site for molecular docking and dynamics tests between SARMs and AR. Given the similar reactivity observed in CAV1 across all three structures, the 1T7T conformation will be utilized due to its higher resolution in the crystallized model.

#### 3.2.2 5α-Reductase II (5αRII)

To investigate the reactivity of 5αRII cavities, we analyzed two crystallized structures: one from the PDB, designated as 7BW1, and another from SSMA, designated as ma-ib3wq. For each protein model, we identified the most reactive cavity sites (CAVs) (Table S2, available in the Supporting Information).

In the 7BW1 model (Figure 4A), eight CAVs were identified. Among these, CAV1 demonstrated the highest druggability, with a peak p*K*_d_ of 10.09, an average p*K*_d_ of 6.91, and a DrugScore of 2749.00, classifying it as highly druggable (++). CAV2 also displayed significant druggability with a peak p*K*_d_ of 9.15, an average p*K*_d_ of 5.75, and a DrugScore of 1151.00, indicating medium druggability (++). In contrast, the other cavities (CAV3 to CAV8) showed considerably lower druggability with negative DrugScores and reduced p*K*_d_ values, indicating poor druggability (−). For the ma-ib3wq structure (Figure 4B), 11 CAVs were identified. CAV1 was again notable, with a peak p*K*_d_ of 11.19, an average p*K*_d_ of 6.45, and a DrugScore of 2503.00, marking it as a highly druggable site (++). Interestingly, CAV6, with a peak p*K*_d_ of 6.99 and an average p*K*_d_ of 5.02, had a DrugScore of 187, suggesting medium druggability (+) but with potential challenges in binding efficiency. The remaining CAVs in this structure (CAV2, CAV3, CAV4, CAV5, and CAV7-CAV11) demonstrated weak druggability with negative DrugScores and lower p*K*_d_ values.

**Figure 4:**
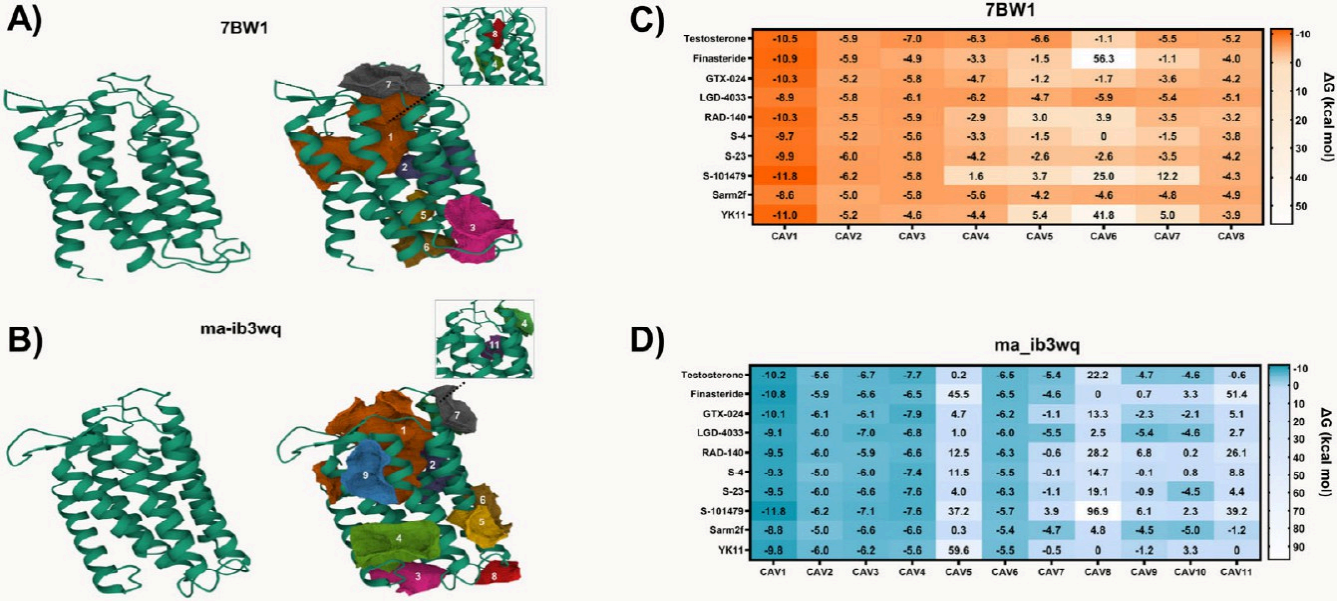
Analysis of the most reactive cavities (CAV) in different 5αRII species (A–B), with data obtained using the CavityPlus Web server. Molecular docking was used to analyze potential affinity between the identified cavities in each 5αRII species and SARMs (C–D), with data obtained through AutoDockVina and Discovery Studio Visualizer.

Molecular docking analyses demonstrated that the highest affinities for testosterone, finasteride, and SARMs were observed primarily in the CAVs exhibiting the highest druggability across the 5αRII conformations tested. Notably, CAV1 consistently showed the strongest interactions with the control molecules, testosterone, and finasteride. Specifically, Δ*G*_bind_ for testosterone binding to CAV1 was −10.5 kcal/mol in the 7BW1 model and −10.2 kcal/mol in the ma-ib3wq model. For finasteride, the binding energies were −10.9 kcal/mol in 7BW1 and −10.8 kcal/mol in ma-ib3wq. Both models, 7BW1 (Figure 4C) and ma-ib3wq (Figure 4D), highlighted CAV1 as the most reactive and druggable site, showing consistently lower binding free energy values in all molecules. Among the SARMs tested, GTX-024, RAD-140, and YK11 demonstrated strong binding affinities at the 5αRII site, with Δ*G*_bind_ values ranging from −10.1 to −10.3 kcal/mol for GTX-024, −9.5 to −10.3 kcal/mol for RAD-140, −9.3 to −9.7 kcal/mol for S-4, and −9.8 to −11.0 kcal/mol for YK11. Of particular note, S-101479 displayed the highest binding affinity with CAV1, with Δ*G*_bind_ −11.8 kcal/mol in both models.

The consistent findings across different 5αRII conformations suggest that targeting CAV1 could be a viable strategy for developing effective modulators. Consequently, CAV1 will be prioritized for further molecular docking and dynamics studies involving SARMs and 5αRII, with the 7BW1 conformation chosen for these subsequent analyses due to its higher resolution.

### 3.3 Interactions of SARMs and Analogs with AR Active Site and 5αRII in Molecular Docking Simulations

#### 3.3.1 Androgen Receptor (AR)

To conduct a comprehensive analysis of the principal interactions of SARMs with the AR in molecular docking studies, we utilized the crystallographic model 17T7, focusing on the primary site of the highest chemical reactivity as determined through detailed cavity mapping. DHT served as the reference ligand, given its role as the endogenous ligand with the highest androgenic potential upon AR binding,^59,60^ providing a control for the comparative analysis of SARMs-AR interactions. Within the AR, residues such as Asn705, Glu711, Arg752, and Thr877 were identified as critical determinants of high-affinity ligand binding, functioning as predictive markers in our docking simulations.^18,61^

In the initial analysis of the AR-DHT complex (Figure 5A; Table S3 [available in Supporting Information]), we observed the formation of robust hydrogen bonds, with Arg752 engaging the O3 ketone at the 3-position of the steroid nucleus in DHT (2.49 Å). This interaction underscores the critical role of the steroidal carbonyl group in stabilizing the AR-DHT complex through a strong electrostatic contribution. Additionally, Asn705 of AR established a hydrogen bond with the O17 hydroxyl group of DHT (2.29 Å), further reinforcing the binding interaction through the formation of a polar network within the ligand-binding domain. van der Waals interactions were identified between Gln711 (7.82 Å) and Thr877 (4.03 Å) of AR with DHT, highlighting the importance of noncovalent forces in maintaining the structural integrity of the complex. Of particular note, alkyl interactions were detected between Leu704 (5.20 Å) of AR and the hydrophobic moieties of DHT, contributing to the stabilization of the steroidal core within the AR binding pocket.^62,63^ Upon examining the AR-GTX-024 complex (Figure 5B; Table S3 [available in Supporting Information]), we identified significant van der Waals interactions between Asn705 (6.75 Å) and Gln711 (11.04 Å) of AR with GTX-024. Although the Arg752 residue did not participate in any direct interactions with GTX-024, a noteworthy carbon–hydrogen bond was observed between Thr877 (2.67 Å) and the O4 atom of GTX-024. This interaction suggests a possible electrostatic contribution that compensates for the lack of direct interaction with Arg752, a residue typically involved in strong binding.

**Figure 5:**
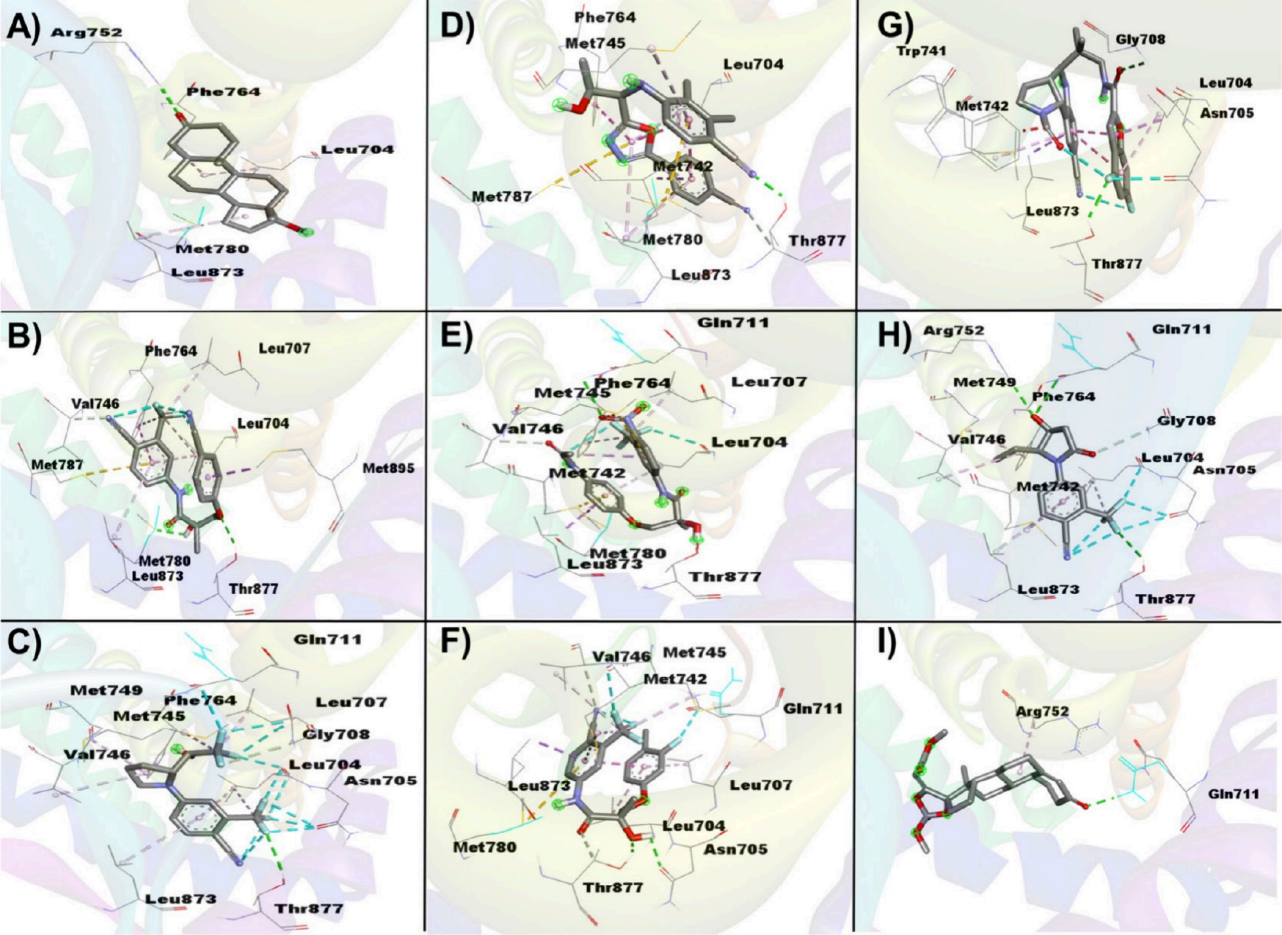
Interaction between AR (CAV1) with SARMs on molecular docking studies. (A) DHT-AR complex; (B) GTX-024-AR complex; (C) LGD-4033-AR complex; (D) RAD-140-AR; (E) S-4-AR complex; (F) S-23-AR complex; (G) S-102479-AR complex; (H) Sarm2f-AR complex; (I) YK11-AR complex. Data obtained using the software AutoDockVina, AutoDockTools and Discovery Studio Visualizer.

Further analysis of the AR-LGD-4033 interaction (Figure 5C; Table S3 [available in Supporting Information]) revealed strong halogen interactions, particularly involving fluorine atoms, with Asn705 (3.54 Å) and Gln711 (3.17 Å) of AR interacting with F10 and F23 of LGD-4033, respectively. The strong electronegativity of the fluorine atoms likely enhances the binding affinity through dipole interactions. Additionally, hydrogen bonding was observed between Thr877 (2.97 Å) of AR and F10 of LGD-4033, suggesting competitive interactions with the Asn705 residue. The closer proximity of the Thr877-F10 interaction indicates a potential preference for this interaction over the Asn705-F10 interaction, indicating a prioritized binding preference within the AR-LGD-4033 complex.

In the AR-RAD-140 complex (Figure 5D; Table S3 [available in Supporting Information]), van der Waals interactions were observed between Asn705 (7.05 Å), Gln711 (13.84 Å), and Arg752 (7.76 Å) of AR with RAD-140. Moreover, a hydrogen bond was identified between Thr877 (2.19 Å) of AR and the N1 atom of RAD-140, reflecting the critical role of hydrogen bonding in ligand stabilization. Similar to the AR-DHT complex, alkyl interactions between Leu704 (5.04 Å) of AR and the aromatic ring of RAD-140 were observed, ensuring the stability of other critical residues within the binding pocket. Analysis of the AR-S-4 complex (Figure 5E; Table S3 [available in Supporting Information]) revealed van der Waals interactions between Asn705 of AR and S-4 (11.98 Å), along with significant hydrogen bonds at Gln711 (2.92 Å) and Thr877 (1.41 Å) with the O10 and O19 atoms of S-4, respectively. These interactions suggest a dual mode of binding, with van der Waals forces providing a broad stabilizing effect, and hydrogen bonds offer specific directional stabilization.

In the AR-S-23 complex (Figure 5F; Table S3 [available in Supporting Information]), van der Waals interactions were identified between Arg752 of AR and S-23 (10.84 Å). Hydrogen bonds were also observed, with Asn705 (2.49 Å) and Gln711 (3.27 Å) of AR interacting with the H27 and O11 atoms of S-23, respectively. Notably, strong halogen interactions were identified between Thr877 (2.60 Å) of AR and F28 of S-23, indicating the critical role of halogen bonding in the specificity of ligand recognition. In the AR-S-101479 complex (Figure 5G; Table S3 [available in Supporting Information]), van der Waals interactions were observed between Gln711 (8.02 Å) and Arg752 (10.02 Å) of AR with S-101479.

Additionally, halogen (fluorine) interactions were detected at Asn705 (2.79 Å) of AR, along with hydrogen bonds involving Thr877 (2.38 Å) and F17 of S-101479. These interactions suggest a complex interplay between halogen bonding and hydrogen bonding in determining the binding orientation and affinity of S-101479 for the AR binding site. In the AR-Sarm2f complex (Figure 5H; Table S3 [available in Supporting Information]), halogen interactions were observed between Asn705 (2.87 Å) of AR and fluorine atoms in Sarm2f. Hydrogen bonds were also noted, with Gln711 (3.23 Å) and Arg752 (2.56 Å) of AR interacting with the O4 atom of Sarm2f. The proximity of Arg752 to Sarm2f suggests a preferential binding interaction, likely due to steric and electrostatic factors. Additionally, Thr877 (2.97 Å) of AR interacted with F1 of Sarm2f through hydrogen bonding, indicating a strong stabilizing interaction. Finally, in the AR-YK11 complex (Figure 5I; Table S3 [available in Supporting Information)), significant interactions were observed, with Gln711 (2.14 Å) of AR engaging in hydrogen bonding with the O3 ketone of YK11. Alkyl interactions were also observed between Arg752 (4.96 Å) of AR and the steroid nucleus of YK11, emphasizing the importance of hydrophobic forces in the stabilization of steroidal ligands within the AR binding pocket, similar showed by AR-DHT complex.

#### 3.3.2 5α-Reductase II (5αRII)

To investigate the potential interactions between 5αRII and SARMs, we conducted a molecular docking study using crystallographic model 7BW1. The docking was focused on the most reactive active site, as identified through cavity search analysis. Testosterone and finasteride were used as reference ligands for 5αRII interaction. We closely monitored the interactions between SARMs and key residues within the 5αRII active site, specifically Glu57, Phe223, and Leu224.

In the 5αRII-testosterone complex (Figure 6A; Table S3 [available in Supporting Information]), we identified π-sigma interactions between Phe223 and the C3–C4 bonds of the steroidal nucleus of testosterone (3.76 Å), which is crucial for the conversion of testosterone to DHT, mediated by the cofactor NADPH. Additionally, van der Waals interactions were observed between the steroidal nucleus and Glu57 (10.71 Å) and Phe223 (6.77 Å). For the 5αRII-finasteride complex (Figure 6B; Table S3 [available in Supporting Information)), π-alkyl interactions were noted between Phe223 and the tertiary amine of finasteride (3.76 Å). Moreover, Glu57 and Phe224 formed van der Waals interactions with finasteride (7.08 Å and 6.77 Å, respectively).

**Figure 6:**
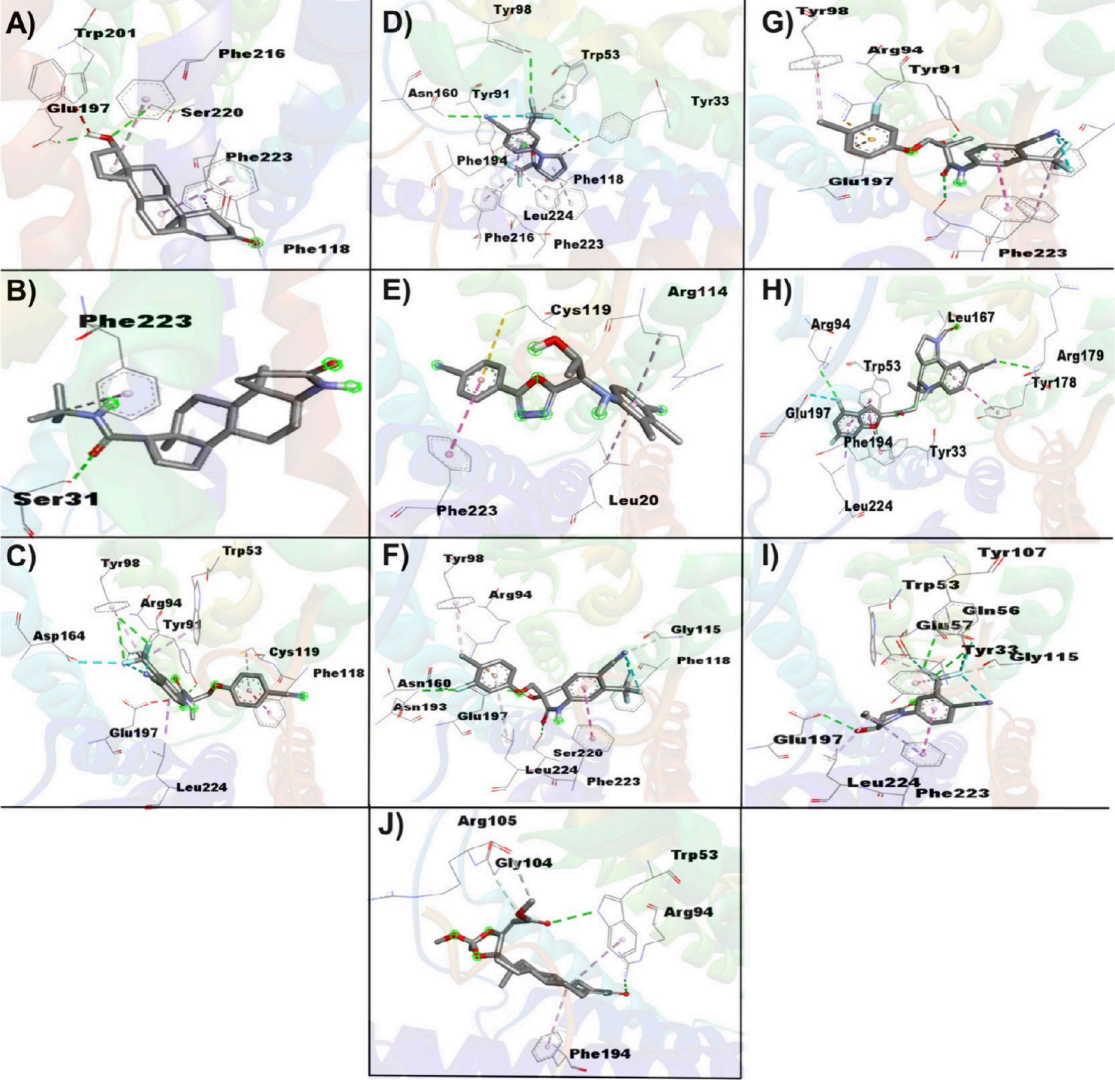
Interaction between 5αRII (CAV1) with SARMs on molecular docking studies. (A) Testosterone-5αRII complex; (B) Finasteride-5αRII complex (C) GTX-024–5αRII complex; (D) LGD-4033–5αRII complex; (E) RAD-140–5αRII; (F) S-4–5αRII complex; (G) S-23–5αRII complex; (H) S-102479–5αRII complex; (I) Sarm2f-5αRII complex; (J) YK11–5αRII complex. Data obtained using the software AutoDockVina, AutoDockTools and Discovery Studio Visualizer.

In the 5αRII-GTX-024 complex (Figure 6C; Table S3 [available in Supporting Information]), van der Waals interactions were detected between Glu57 and Phe223 with GTX-024 (9.07 and 7.56 Å, respectively). The trifluoromethyl aniline group of GTX-024 established a π-sigma interaction with Leu224 (3.77 Å). For the 5αRII-LGD-4033 complex (Figure 6D; Table S3 [available in Supporting Information]), van der Waals interactions were identified with Glu57 (6.25 Å), alongside significant π-alkyl interactions between Phe223 and the cyclopentane ring of LGD-4033 (4.21 Å), and π-sigma interactions between the aromatic ring of the trifluoromethyl aniline and Leu224 (3.67 Å). In the 5αRII-RAD-140 complex (Figure 6E; Table S3 [available in Supporting Information]), van der Waals interactions were observed with Glu57 (7.23 Å) and π-π T-shaped interactions between Phe223 and the phenylcyclopentane group of RAD-140 (5.20 Å). RAD-140 did not interact with Leu224. For the 5αRII-S-4 complex (Figure 6F; Table S3 [available in Supporting Information]), van der Waals interactions were seen between Glu57 and S-4 (8.54 Å), along with crucial π-π T-shaped interactions between Leu224 and Phe223 and the aromatic rings of S-4 (5.06 and 5.27 Å, respectively).

In the 5αRII-S-23 complex (Figure 6G; Table S3 [available in Supporting Information]), π-π T-stacked interactions were detected between Phe223 and the aromatic ring associated with the trifluoromethyl aniline group of S-23 (4.44 Å), as well as π-alkyl interactions between the fluorobenzene moiety of S-23 and Leu224 (5.41 Å). No interactions were observed between Glu57 and S-23. For the 5αRII-S-101479 complex (Figure 6H; Table S3 [available in Supporting Information]), no interactions were identified between Glu57 and S-101479. However, π-sigma interactions were observed between Leu224 and the difluorobenzene group of S-101479 (3.70 Å), along with van der Waals interactions between Phe223 and S-101479 (7.85 Å). In the 5αRII-Sarm2f complex (Figure 6I; Table S3 [available in Supporting Information]), halogen (fluorine) bonds were observed between F2 of Sarm2f and Glu57 (3.13 Å), along with π-π stacked interactions between the aromatic ring of Sarm2f and Phe223 (4.43 Å). Significant alkyl interactions were also noted between the cyclopentane groups of Sarm2f and Leu224 (4.50 Å). Finally, in the 5αRII-YK11 complex (Figure 6J; Table S3 [available in Supporting Information]), van der Waals interactions were observed between the steroidal nucleus of YK11 and all three critical residues, Glu57, Phe223, and Leu224 (10.11 Å, 7.94 Å, and 6.15 Å, respectively).

### 3.4 Interactions of SARMs and Analogs with AR Active Site and 5αRII in Molecular Dynamics Simulations

#### 3.4.1 Androgen Receptor (AR)

The *R*_g_ is crucial for assessing the conformational stability and spatial distribution of ligands complexed with the AR (Figure 7A). DHT, with an *R*_g_ of 17.83 Å, serves as a compact steroidal reference. GTX-024, with an *R*_g_ of 18.24 Å, shows slightly more expansion due to its nonsteroidal nature and flexible structure. LGD-4033 has the highest *R*_g_ = 18.46 Å, indicating significant flexibility and spatial extension, while RAD-140, with an *R*_g_ = 18.14 Å, is more compact. S-4 and S-23, with *R*_g_ values of 18.17 Å and 18.31 Å, respectively, differ in compactness due to structural variations. S-101479, with an *R*_g_ 18.26 Å, shows moderate flexibility, while Sarm2f with a *R*_g_ = 18.13 Å, is more compact. YK11, with an *R*_g_ of 17.91 Å, is closest to DHT in compactness, ensuring tight interaction with AR.

**Figure 7:**
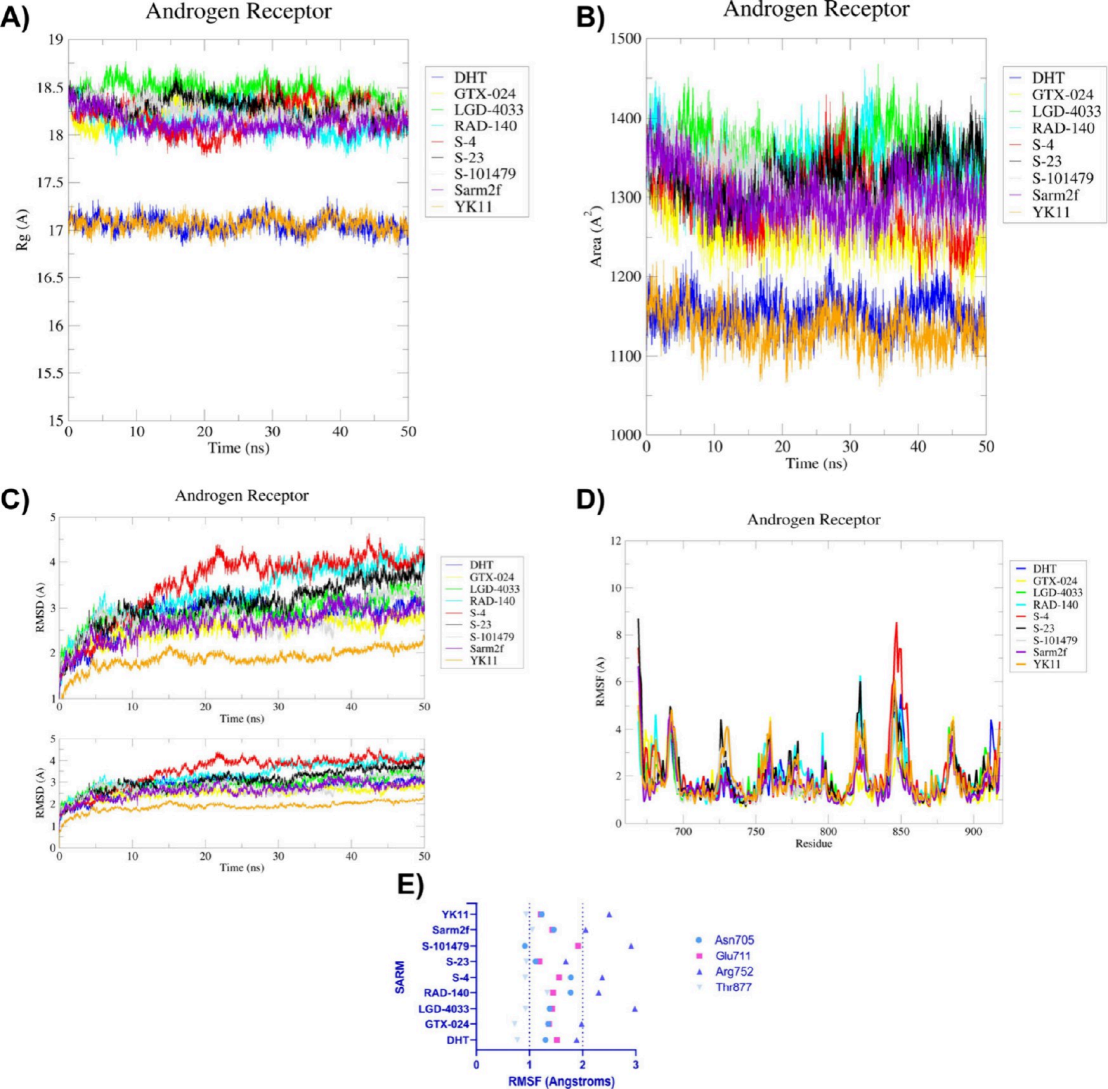
Molecular dynamics simulation of the backbone for the complexes between SARMs-AR over 50 ns, represented by (A) Radius of Gyration (*R*_g_) trajectories; (B) Solvent Accessible Surface Area (SASA) trajectories; (C) Root-Mean-Square Deviation (RMSD) plots; (D) Root Mean Square Fluctuation (RMSF) plots; (E) The spatial arrangement of key residues in AR function, along with the distances between the backbone structures and these residues (scatter dot plot).

The SASA is crucial for evaluating molecular surface exposure in aqueous environments, influencing receptor interactions and solubility (Figure 7B). DHT, as the control, has a SASA of 1221 Å^2^, reflecting its compact steroidal structure. GTX-024, with a SASA of 1345 Å^2^, shows slightly greater solvent exposure due to its nonsteroidal and flexible structure. LGD-4033 has the highest SASA at 1355 Å^2^, indicating significant surface exposure due to its bulky aromatic system. RAD-140, with an SASA of 1337 Å^2^, is slightly more compact. S-4 and S-23, with SASA values of 1299 and 1323 Å^2^, respectively, differ in solvent exposure due to variations in side chain length and flexibility. S-101479, at 1306 Å^2^, demonstrates moderate surface exposure, while Sarm2f, with a SASA of 1302 Å^2^, has a more compact structure. Finally, YK11, with a SASA of 1257 Å^2^, shows a structure closer to that of DHT, reflecting its steroidal backbone and resulting in reduced solvent exposure.

The RMSD reveals the conformational stability of ligands bound to AR (Figure 7C). Lower RMSD values suggest more stable and consistent binding poses, while higher values indicate greater flexibility or structural variation. DHT, with an RMSD of 2.80 Å, serves as a control and demonstrates a stable, rigid steroidal framework with minimal conformational fluctuations. GTX-024 shows a slightly lower RMSD of 2.51 Å, reflecting stable interaction with the AR despite its nonsteroidal structure and flexible alkyl chains. LGD-4033, with an RMSD of 2.89 Å, exhibits increased conformational flexibility due to its bulkier aromatic rings, leading to more dynamic interactions. RAD-140 has the highest RMSD of 3.35 Å, indicating significant structural variability likely due to its nonsteroidal nature and the arrangement of its functional groups. S-4 and S-23 show RMSD values of 3.57 Å and 3.07 Å, respectively, with S-4 displaying a higher RMSD and thus less stability compared to S-23, due to their larger hydrophobic regions and nonsteroidal backbones. S-101479, with an RMSD of 2.74 Å, maintains moderate stability with some flexibility due to its complex structure and additional substituents. Sarm2f, having an RMSD of 2.89 Å, exhibits a compact and stable conformation with reduced structural variation. YK11, with the lowest RMSD of 1.75 Å, demonstrates the highest stability and minimal conformational fluctuations, consistent with its steroidal backbone and structural similarity to DHT.

The RMSF data indicates the flexibility of key residues in the AR across various ligands (Figure 7D, 7E). For DHT, RMSF values are 1.301 Å for Asn705, 1.515 Å for Glu711, 1.885 Å for Arg752, and 0.771 Å for Thr877. GTX-024 shows similar patterns with slightly higher values at Arg752 (1.981 Å) and lower values at Thr877 (0.718 Å). LGD-4033 displays an increased fluctuation, particularly at Arg752 (2.983 Å). RAD-140 and S-4 have even greater flexibility, with RAD-140 showing the highest RMSF values at Arg752 (2.301 Å) and Thr877 (1.335 Å) and S-4 also showing notable fluctuation at Arg752 (2.369 Å). S-23 has lower RMSF values, indicating more stability, especially at Arg752 (1.684 Å). S-101479 exhibits unusually high RMSF values, particularly at Thr877 (3.913 Å), indicating a significant instability. Sarm2f shows moderate flexibility, while YK11 has relatively stable RMSF values with the highest variability at Arg752 (2.502 Å), reflecting some fluctuation but overall stability in binding.

#### 3.4.2 5α-Reductase II (5αRII)

The *R*_g_ of 5αRII is depicted below (Figure 8A). Testosterone has an *R*_g_ of 18.79 Å, indicative of a compact steroidal structure. Finasteride, with a notably higher *R*_g_ of 19.10 Å, suggests a more constrained conformation. GTX-024, however, stands out with an *R*_g_ of 19.19 Å, indicating considerable spatial extension attributed to its nonsteroidal and flexible nature. LGD-4033, with an *R*_g_ of 18.40 Å, remains relatively compact, while RAD-140 shows an *R*_g_ of 19.18 Å, reflecting slight expansion. S-4, with an *R*_g_ of 18.33 Å, indicates a more compact structure, while S-23, at 19.04 Å, reveals significant expansion due to structural flexibility. S-101479, with an *R*_g_ of 17.95 Å, shows moderate spatial extension, and Sarm2f exhibits an *R*_g_ of 19.03 Å, indicating good flexibility. YK11, with an *R*_g_ of 18.25 Å, maintains a compact conformation, similar to testosterone.

**Figure 8:**
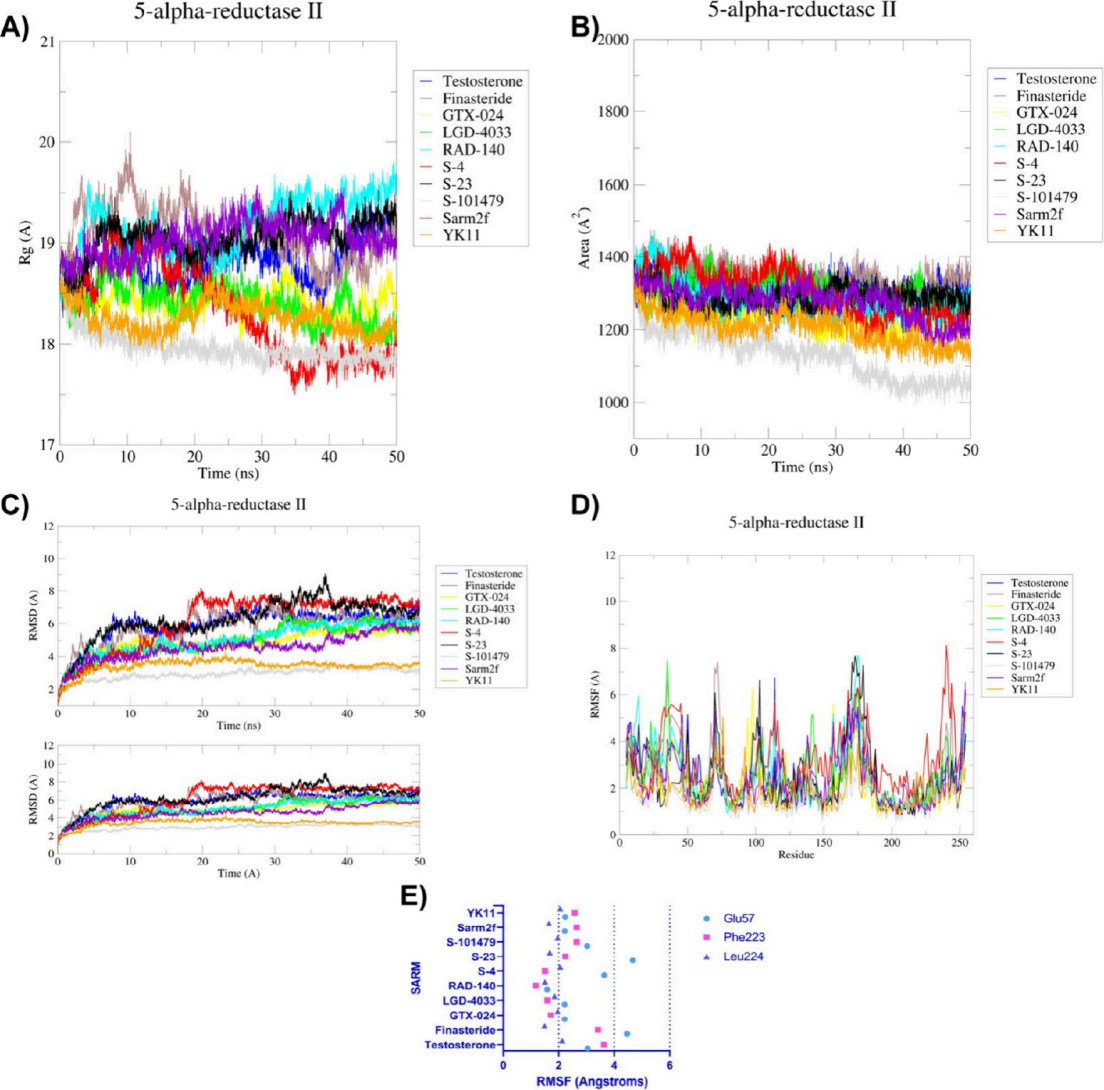
Molecular dynamics simulation of the backbone for the complexes between SARMs-5αRII over 50 ns, represented by (A) Radius of Gyration (*R*_g_) trajectories; (B) Solvent Accessible Surface Area (SASA) trajectories; (C) Root-Mean-Square Deviation (RMSD) plots; (D) Root Mean Square Fluctuation (RMSF) plots; (E) The spatial arrangement of key residues in 5αRII function, along with the distances between the backbone structures and these residues (scatter dot plot).

The SASA of 5αRII is depicted below (Figure 8B). Testosterone exhibits an SASA of 1309 Å^2^, reflecting its compact structure. Finasteride, with a SASA of 1349 Å^2^, shows slightly greater solvent exposure, while GTX-024, at 1300 Å^2^, indicates moderate surface exposure. LGD-4033 SASA of 1310 Å^2^ suggests stable exposure, whereas RAD-140, with a lower SASA of 1283 Å^2^, demonstrates a more compact structure. S-4 and S-23 present SASA values of 1304 and 1288 Å^2^, respectively, with S-23 indicating slightly less solvent exposure. S-101479, with an SASA of 1128 Å^2^, shows reduced exposure, while Sarm2f, at 1277 Å^2^, indicates a more moderate surface exposure. Finally, YK11, with a SASA of 1203 Å^2^, reflects a structure that is less exposed to solvent, aligning it closely with the compact nature of testosterone.

The RMSD of 5αRII is depicted below (Figure 8C). Testosterone, with an RMSD of 5.88 Å, establishes a reference point, demonstrating significant flexibility characteristic of its steroidal nature. Finasteride closely follows with an RMSD of 5.76 Å, suggesting stable yet dynamic binding interactions. GTX-024 exhibits a lower RMSD of 4.82 Å, reflecting a more consistent binding pose despite its nonsteroidal structure. LGD-4033, at 4.94 Å, indicates a moderate degree of flexibility, while RAD-140, with an RMSD of 5.07 Å, also demonstrates some structural variability. S-4 presents the highest RMSD at 6.16 Å, indicating substantial fluctuations in its binding conformation, closely followed by S-23 at 6.22 Å, which further suggests increased instability. In contrast, S-101479 shows a markedly lower RMSD of 2.96 Å, indicating excellent stability and minimal conformational variations. Sarm2f, with an RMSD of 4.57 Å, reflects a stable interaction, while YK11, at 3.44 Å, combines significant stability with moderate flexibility.

The RMSF of 5αRII are depicted below (Figure 8D, 8E). For testosterone, the RMSF values for Glu57, Phe223, and Leu224 are 3.043 3.634, and 2.128 Å, respectively, indicating moderate to high flexibility. Finasteride shows a higher fluctuation, particularly at Glu57 (4.464 Å), suggesting significant dynamic interactions. GTX-024, with RMSF values of 2.215 Å for Glu57, 1.715 Å for Phe223, and 1.962 Å for Leu224, indicates relatively lower flexibility, reflecting stable binding. LGD-4033 mirrors these results with an RMSF of 2.215 Å for Glu57, 1.590 Å for Phe223, and 1.850 Å for Leu224. RAD-140 demonstrates lower RMSF values, with the highest fluctuation at Glu57 (1.581 Å), reflecting its more rigid binding conformation. S-4 and S-23 exhibit greater flexibility, especially with S-23 showing an RMSF of 4.668 Å at Glu57. S-101479 maintains a moderate flexibility, particularly at Phe223 (2.648 Å). Sarm2f shows consistent RMSF values, while YK11 displays moderate fluctuations with the highest value at Glu57 (2.231 Å), indicating a generally stable binding interaction with some variability.

### 3.5 Principal Component Analysis (PCA) of in AR and 5αRII Proteins

The principal component analysis (PCA) of the backbone atoms for Sarm2f, YK11, DHT, and finasteride aimed to capture dominant motion patterns over the 50 ns simulation (Figure 9). PCA, a dimensionality reduction method, projects data onto orthogonal vectors, known as principal components (PCs), which represent the highest variance within the data set. Comparing the results, DHT and YK11 (Figure 9A, 9C) display broader distributions along PC1 and PC2, indicating considerable conformational flexibility and possible dynamic adaptations within the AR. In contrast, Sarm2f (Figure 9B) shows a more compact distribution along the PCs, suggesting a more stable conformation with limited structural variation. For 5αRII, there is a notable difference between finasteride (Figure 9D), which exhibits a more diffuse spread, and Sarm2f and YK11 (Figure 9E, 9F), which display tighter clustering along the first two components, indicating stable conformations with high affinity for 5αRII. This conformational stability, evidenced by close clustering along the PCs, likely arises from optimized molecular configurations, where halogenated and aromatic moieties enhance binding energetics while minimizing steric clashes. Therefore, both Sarm2f and YK11 exhibit selective dual binding potential for AR activation and 5αRII inhibition, positioning them as promising candidates for future clinical investigation.

**Figure 9:**
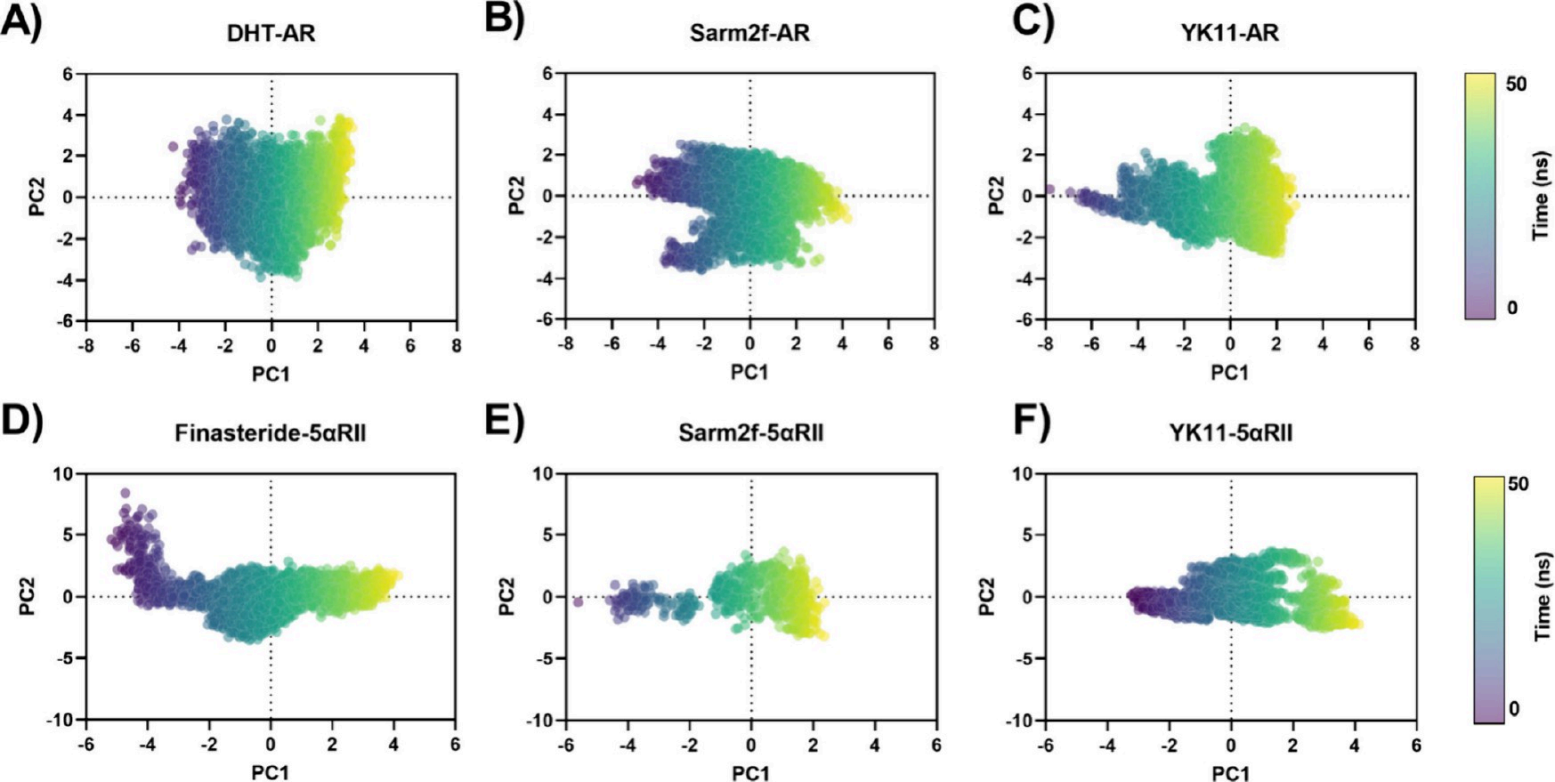
Scatter plots of the first two principal components (PC1 and PC2) thought time frame in nanoseconds. The plots represent the molecular dynamics over 50 ns for the following complexes: (A) DHT-AR, (B) Sarm2f-AR, (C) YK11-AR, (D) Finasteride-5αRII, (E) Sarm2f-5αRII, (F) Sarm2f-5αRII.

### 3.6 Analyzing Sarm2f and YK11 for Enhanced AR and 5αRII Modulation

The unique chemical structures and physicochemical properties of SARMs such as YK11 and Sarm2f enable highly selective interactions with both the AR and 5αRII, enhancing anabolic pathway modulation while minimizing androgenic side effects and off-target activity. YK11 has been shown to directly upregulate AR mRNA expression^64^ and increase AR protein levels *in vivo*.^65^ Our docking and molecular dynamics simulations mirror these findings, revealing strong binding affinities and remarkably stable AR–YK11 complexes. Sarm2f has demonstrated higher AR activation than testosterone and potency comparable to GTX-024 in AR-expressing cells from rodents, nonhuman primates, and humans.^66^ These outcomes are consistent with our computational observations, in which Sarm2f exhibited robust docking scores and sustained receptor engagement throughout the simulation period. To elucidate their binding affinities, interaction profiles, and conformational stabilities, we employed an integrative computational approach encompassing molecular docking, DFT calculations, and molecular dynamics simulations, with emphasis on YK11 and Sarm2f due to their greater interaction profiles in AR and 5αRII proteins.

Sarm2f exhibited a high stable binding profile, engaging in optimal interactions with critical residues within the AR, such as Asn705, Glu711, Arg752, and Thr877.^17,18,67^ Its fluorinated groups confer strong electron-withdrawing capabilities, augmenting hydrogen bond interactions through dipole induction.^68,69^ This localized negative potential enhances binding affinity through polar interactions, aligning with halogen bonding theory in which the high electronegativity of fluorine induces a partial negative charge that supports stable C–F···H interactions with donor residues,^70,71^ including critical residues, such as Thr877 in AR. Additionally, Sarm2f structure facilitates π-stacking interactions with hydrophobic AR residues, yielding a compact and energetically favorable binding conformation. Sarm2f interactions with 5αRII, specifically at key residues Glu57, Phe223, and Leu224, stabilize the complex through π-stacking and π–σ interactions within hydrophobic pockets. The rigid aromatic scaffold of Sarm2f imposes steric constraints that limit substrate flexibility, thereby enhancing the stability of the ligand–receptor complex with minimal conformational fluctuations. Its low RMSD values and compact *R*_g_ within the 5αRII binding pocket indicate hydrophilicity and solvation dynamics, which are essential for preserving bioactivity in physiological conditions.^66,72^

Upon analysis, YK11 exhibits distinct binding behaviors with both AR and 5αRII, driven by its steroidal core structure, which confers significant androgenic activity.^6^ YK11 molecule includes significant nucleophilic regions, with electrostatic potential data revealing negative potentials around oxygen atoms, O14 and O15. These regions enhance the AR binding affinity, contributing to YK11 dual activity as an AR agonist and a myostatin inhibitor.^59,73^ The inhibition of myostatin promotes muscle hypertrophy, adding a distinctive feature to YK11 anabolic profile.^6^ While the precise molecular mechanisms underlying this inhibition remain poorly characterized and likely involve intricate signaling pathways, it is important to emphasize that YK11 is valued not only for its direct anabolic effects but also for its potential to modulate myostatin activity. Although this article does not delve into this mechanism in depth, the role of YK11 in myostatin inhibition warrants further investigation.

Within the AR-YK11 complex, strong binding interactions were observed, notably a hydrogen bond between Gln711 of AR and the O3 ketone of YK11. Additionally, alkyl interactions between Arg752 and the steroidal core of YK11 emphasize the role of hydrophobic forces in ligand stabilization within the AR binding pocket, which is akin to interactions observed in the AR-DHT complex. YK11 also displayed a low SASA, which is indicative of a conformation closely resembling DHT with reduced solvent exposure, which likely enhances AR affinity. Additionally, compact YK11 *R*_g_ aligns closely with that of testosterone, indicating a stable binding conformation that optimally positions YK11 within the AR site, supporting selective AR modulation while minimizing off-target interactions. The presence of functional groups, such as the 1-methoxyethylidene moiety and the C21 carboxylate methyl group, modulates lipophilicity and contributes to metabolic stability and bioavailability, thereby enhancing YK11 pharmacological potential.^6,73^ YK11 achieves a conformational balance that limits displacement during dynamic simulation. The slight flexibility of YK11, evidenced by low RMSF fluctuations of specific residues like Arg752, enhances its interaction potential within the AR binding site, albeit with a small trade-off in selectivity when compared to the more rigid Sarm2f.

In the 5αRII-YK11 complex, van der Waals interactions were observed between the steroidal core of YK11 and residues Glu57, Phe223, and Leu224. These interactions likely contribute to the relatively weak binding affinity observed in docking studies, indicating that van der Waals forces play a limited role in the binding. However, molecular dynamics simulations revealed low RMSD values, with YK11 maintaining a high stability and minimal conformational fluctuations due to the intrinsic rigidity of its steroidal backbone. This stable conformation within the binding pocket suggests that despite the initial lower binding affinity observed in docking, YK11 adopts a stable orientation that minimizes displacement during dynamic simulations.

The interplay of these structural characteristics demonstrates that while both Sarm2f and YK11 are potent SARMs, each exhibits distinct conformational dynamics and binding affinities, conferring unique advantages. The rigidity of Sarm2f ensures greater stability and selectivity, while the flexibility of YK11, coupled with its structural similarity to steroid hormones, grants it enhanced binding versatility. These observations highlight the critical role of specific chemical moieties in modulating receptor affinity, conformational stability, and selective interactions with AR and 5αRII. This insight paves the way for the design of more targeted and effective SARM-based therapies.

### 3.7 Study Limitations

This study primarily relies on in silico methodologies to investigate the molecular interactions and conformational dynamics of SARMs with androgenic and steroidogenic targets. Computational approaches such as docking, DFT, and molecular dynamics simulations offer valuable and mechanistically detailed insights that are difficult to obtain through early stage experimental setups. However, we recognize that these methods, while highly informative, do not fully replicate the complexity of biological systems. Thus, the findings presented here should be interpreted as predictive rather than confirmatory. Nonetheless, by establishing a robust computational framework for exploring the activity of SARMs on androgen receptors and steroidogenic targets such as 5αRII, this work aims to provide a solid foundation for future investigations. Given the limited pharmacological data currently available for many SARMs (despite their widespread off-label use), preliminary studies such as these are essential to guide subsequent experimental efforts and accelerate progress in this underexplored field.

Also, although additional aspects such as ADMET profiling and interactions with other steroidogenic enzymes (e.g., aromatase, 5α-reductase type I, and estrogen receptors) were considered during the study’s conceptualization, they were deliberately set aside to preserve analytical depth and coherence to explore AR and 5αRII interactions. We acknowledge that the absence of experimental validation and extended pharmacokinetic profiling constitutes a limitation; however, given the scarcity of mechanistic data available for many SARMs, this focused investigation offers meaningful insights and a strategic entry point for future research.

## 4 Concluding Remarks

This study highlights the promising effects of Sarm2f and YK11 as potent SARMs, characterized by their strong binding affinity and selectivity for AR and 5αRII. Structural modifications significantly enhance their interaction profiles, establishing them as pharmacologically active compounds. Utilizing computational methods, such as DFT calculations, molecular docking, and molecular dynamics simulations, has yielded valuable insights for optimizing their pharmacological properties. The main goal is to improve SARM selectivity and specificity, which is vital for reducing androgenic side effects while enhancing therapeutic efficacy. Future *in vivo* studies are crucial to validate the pharmacokinetic and biological effects of SARMs, especially Sarm2f and YK11, paving the way for safer and more effective SARMs tailored to clinical needs and advancing treatment for androgen-mediated disorders.

## Supplementary Material



## Data Availability

The supporting data underlying the findings of this study are available from the corresponding author upon reasonable request or can be accessed directly from the public repository (DOI: 10.5281/zenodo.15685141). Additionally, the protein cavity coordinates can be retrieved by entering the respective PDB codes into the CavityPlus Web server (http://www.pkumdl.cn/cavityplus/).

## Notes

Tables S1 and S2: Results of the cavity prediction and druggability for AR proteins (IDs: 1T7T, 2AMA, and 2PIW) and 5αRII proteins (IDs: 7BW1 and ma-ib3wq); Table S3: Details regarding interactions with critical activation residues in molecular docking, in both AR and 5αRII proteins (PDF)
