# Supplementary material for: A SARM a Day Keeps the Weakness Away: A Computational Approach for Selective Androgen Receptor Modulators (SARMs) and Their Interactions with Androgen Receptor and 5‑Alpha Reductase Proteins
Source: ACS Omega. 2025 Jul 18;10(29):31649–67. doi: 10.1021/acsomega.5c02504 (PMC12311730; doi:10.1021/acsomega.5c02504)
Supplement: Supplementary file 1 [file ao5c02504_si_001.pdf]

*Supporting information for*

**"An SARM a day keeps the weakness away: Computational approach of Selective Androgen Receptor Modulators (SARMs) and their interactions with androgen receptor and 5-alpha reductase proteins"**

Mustafa Munir Mustafa Dahleh[a], Silvana Peterini Boeira[a], Hecson Jesser Segat[a], Gustavo Petri Guerra[a], and Marina Prigol[a*]

[a]Laboratory of Pharmacological and Toxicological Evaluations Applied to Bioactive Molecules - LaftamBio - Federal University of Pampa, Itaqui, CEP 97650-000, RS, Brazil.

[*]Corresponding author:
Marina Prigol; E-mail: marinaprigol@gmail.com; TEL: (55) 99644-7424, Laboratory of Pharmacological and Toxicological Evaluations Applied to Bioactive Molecules – LaftamBio – Federal University of Pampa, Itaqui, CEP 97.650-000, RS, Brazil.

**Table S1.** Results of cavity prediction and druggability for AR species

| Protein | Cavity | Max. pKd | Max. Ave. | DrugScore | Druggability* |
|---|---|---|---|---|---|
| 1T7T | #1 | 10.37 | 6.93 | 1392.00 | ++ |
| | #2 | 10.90 | 6.36 | 301.00 | + |
| | #3 | 9.45 | 5.86 | 26.00 | + |
| | #4 | 8.71 | 5.60 | -730.00 | - |
| | #5 | 6.76 | 4.94 | -1427.00 | - |
| | #6 | 6.58 | 4.88 | -1142.00 | - |
| | #7 | 6.47 | 4.84 | -660.00 | - |
| | #8 | 6.30 | 4.78 | -766.00 | - |
| 2AMA | #1 | 10.94 | 6.97 | 1655.00 | ++ |
| | #2 | 10.03 | 6.06 | 39.00 | + |
| | #3 | 9.39 | 5.84 | 81.00 | + |
| | #4 | 7.71 | 5.26 | -784.00 | - |
| | #5 | 7.57 | 5.21 | -1068.00 | - |
| 2PIW | #1 | 10.29 | 6.92 | 1591.00 | ++ |
| | #2 | 9.40 | 5.84 | -460.00 | - |
| | #3 | 8.81 | 5.64 | -71.00 | + |
| | #4 | 8.15 | 5.41 | -747.00 | - |
| | #5 | 7.63 | 5.23 | -218.00 | - |
| | #6 | 6.52 | 4.86 | -980.00 | - |
| | #7 | 6.13 | 4.72 | -1487.00 | - |
| | #8 | 5.98 | 4.67 | -1373.00 | - |

*[++] Strong (≥ 600 DrugScore); [+] Medium (600 > DrugScore ≥ -180); [-] Weak (< -180).

**Table S2.** Results of cavity prediction and druggability for 5αRII species

| Protein | Cavity | Max. pKd | Max. Ave. | DrugScore | Druggability* |
|---|---|---|---|---|---|
| 7BW1 | 1 | 10.09 | 6.91 | 2749.00 | ++ |
| | 2 | 9.15 | 5.75 | 1151.00 | ++ |
| | 3 | 8.24 | 5.44 | -472.00 | - |
| | 4 | 8.06 | 5.38 | -604.00 | - |
| | 5 | 7.43 | 5.16 | -799.00 | - |
| | 6 | 7.34 | 5.14 | -1190.00 | - |
| | 7 | 6.37 | 4.80 | -992.00 | - |
| | 8 | 5.59 | 4.54 | -1410.00 | - |
| ma-ib3wq | 1 | 11.19 | 6.45 | 2503.00 | ++ |
| | 2 | 10.59 | 6.25 | 1492.00 | ++ |
| | 3 | 9.88 | 6.00 | 44.00 | + |
| | 4 | 9.05 | 5.72 | -523.00 | - |
| | 5 | 8.68 | 5.59 | -487.00 | - |
| | 6 | 8.56 | 5.55 | 187.00 | + |
| | 7 | 6.74 | 4.93 | -1059.00 | - |
| | 8 | 6.31 | 4.78 | -1423.00 | |
| | 9 | 6.13 | 4.72 | -1117.00 | - |
| | 10 | 6.08 | 4.71 | -1367.00 | - |
| | 11 | 5.91 | 4.64 | -1376.00 | - |

*[++] Strong (≥ 600 DrugScore); [+] Medium (600 > DrugScore ≥ -180); [-] Weak (< -180).

**Table S3**. Key interacting residues with AR and 5αRI/II in molecular docking studies.

| Compound | AR (Å) | | | | 5αRI (Å) | | 5αRII (Å) | | |
|---|---|---|---|---|---|---|---|---|---|
| | Asn705 | Gln711 | Arg752 | Thr877 | Arg90 | Asn222 | Glu57 | Phe223 | Leu224 |
| DHT | 2.29 | 7.82# | 2.49 | 4.03# | x | x | x | x | x |
| Testosterone | x | x | x | x | 2.79 | 9.00# | 10.71# | 6.77# | 3.76 |

| | | | | | | | | | |
|---|---|---|---|---|---|---|---|---|---|
| Finasteride | x | x | x | x | 2.77 | 6.40# | 7.08# | 3.76 | 6.77# |
| GTX-024 | 6.76# | 11.04# | Ø | 2.67 | 4.51 | 6.03# | 9.07# | 7.56# | 3.77 |
| LGD-4033 | 3.54 | 3.17 | Ø | 2.97 | Ø | 2.82 | 6.25# | 4.21 | 3.67 |
| RAD-140 | 7.05# | 13.84# | 7.76# | 2.19 | 8.35# | Ø | 7.23# | 5.20 | Ø |
| S-4 | 11.98# | 2.92 | Ø | 1.41 | 2.45 | 2.88 | 8.54# | 5.06 | 5.27 |
| S-23 | 2.49 | 3.27 | 10.84# | 2.60 | 9.70# | 2.63 | Ø | 4.44 | 5.41 |
| S-101479 | 2.79 | 8.02# | 10.02# | 2.38 | 3.33 | Ø | Ø | 7.85# | 3.70 |
| Sarm2f | 2.87 | 3.23 | 2.56 | 2.97 | Ø | 2.60 | 3.13 | 4.43 | 4.50 |
| YK11 | Ø | 2.14 | 4.96 | Ø | 3.02 | 7.60# | 10.11# | 7.94# | 6.15# |

x not tested; Ø no interaction; #Van der Waals interaction.
